# Using natural language processing to extract information from clinical text in electronic medical records for populating clinical registries: a systematic review

**DOI:** 10.1093/jamia/ocaf176

**Published:** 2025-10-15

**Authors:** Leibo Liu, Victoria Blake, Matthew Barman, Blanca Gallego, Timothy Churches, Georgina Kennedy, Sze-Yuan Ooi, Geoffrey P Delaney, Louisa Jorm

**Affiliations:** Centre for Big Data Research in Health, University of New South Wales, Sydney, NSW 2052, Australia; Centre for Big Data Research in Health, University of New South Wales, Sydney, NSW 2052, Australia; Centre for Big Data Research in Health, University of New South Wales, Sydney, NSW 2052, Australia; Centre for Big Data Research in Health, University of New South Wales, Sydney, NSW 2052, Australia; Ingham Institute for Applied Medical Research, Liverpool, NSW 2170, Australia; Ingham Institute for Applied Medical Research, Liverpool, NSW 2170, Australia; South Western Sydney Clinical School, University of New South Wales, Sydney, NSW 2052, Australia; School of Clinical Medicine, University of New South Wales, Sydney, NSW 2052, Australia; Prince of Wales Hospital, Randwick, NSW 2031, Australia; Ingham Institute for Applied Medical Research, Liverpool, NSW 2170, Australia; South Western Sydney Clinical School, University of New South Wales, Sydney, NSW 2052, Australia; Centre for Big Data Research in Health, University of New South Wales, Sydney, NSW 2052, Australia

**Keywords:** clinical registries, natural language processing, information extraction, electronic medical records, clinical text

## Abstract

**Objective:**

Clinical registries advance healthcare by tracking patient outcomes and intervention safety. Manually extracting information from clinical text for registries is labor- and resource-intensive and often inaccurate. Therefore, this systematic review aims to evaluate the use and effectiveness of natural language processing (NLP) methods in extracting information from clinical text for populating clinical registries.

**Materials and Methods:**

PubMed, Embase, Scopus, Web of Science, and ACM Digital Library were systematically searched. Studies were included if they used NLP techniques to populate clinical registries. The extracted data included details of the registry, the clinical text, the registry data elements extracted, the NLP methods used, and how their performance was evaluated.

**Results:**

Fifteen articles were included in the review. Since 2020, the use of NLP methods for extracting information to populate clinical registries has been increasing steadily. Initially, rule-based NLP methods dominated the field, but machine learning-based approaches have gradually gained popularity. However, only one of the included studies employed generative large language models (LLMs). The diversity of clinical text and extracted data elements posed challenges to the generalizability of the NLP methods.

**Conclusion:**

To date, the application of NLP methods to clinical text for populating clinical registries has been limited in both the number of published studies and the scope of implementation. The NLP methods used thus far face significant challenges in effectively managing the complexity and diversity of clinical text and data elements. Moreover, the performance of the NLP methods varied significantly. This review underscores the need for a robust and adaptable NLP framework. Generative LLMs may provide direction for future research, but their use must account for challenges such as accuracy, cost, privacy, and limited supporting evidence.

## Introduction

**Figure 1.**
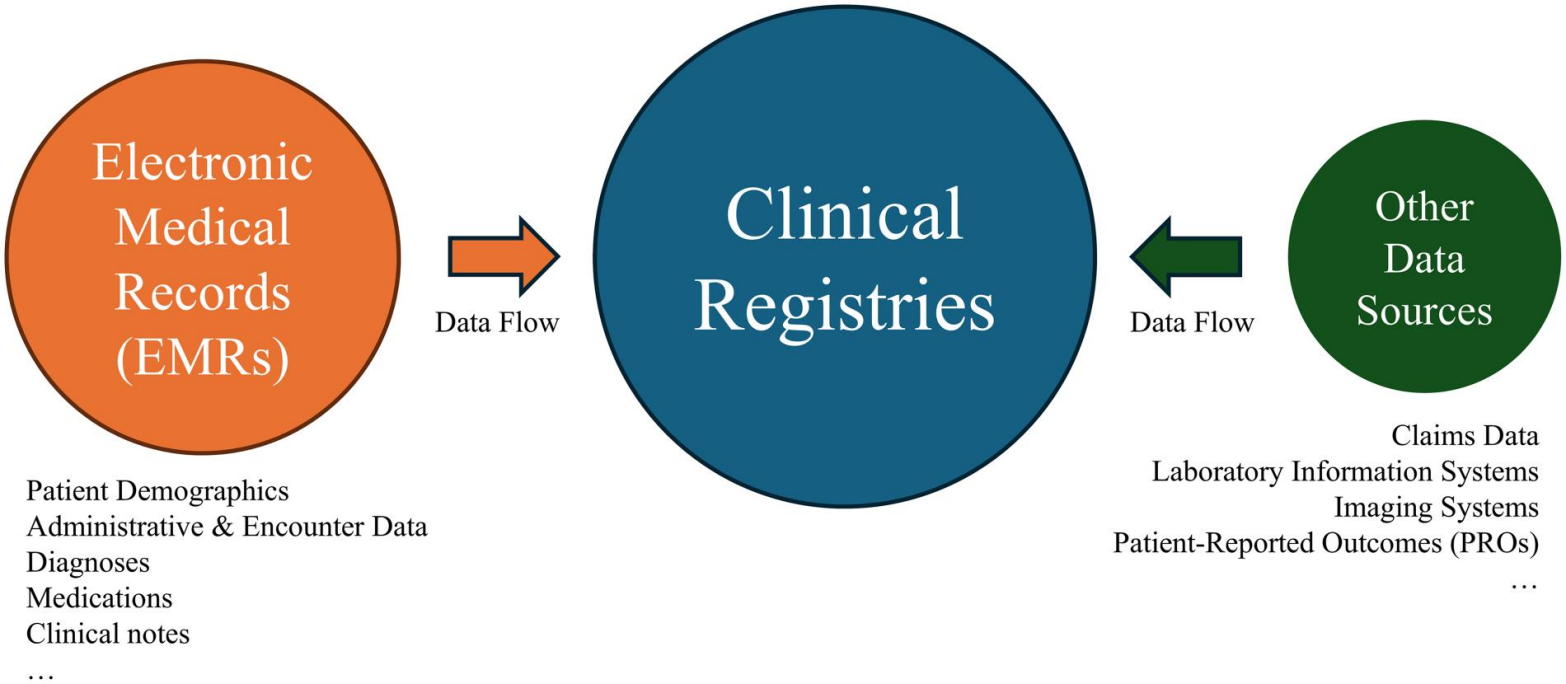
Relationship between electronic medical records (EMRs) and clinical registries.

Clinical registries are organized systems to systematically and comprehensively collect, store, and analyze valuable, real-world, and uniform patient data on an ongoing basis to evaluate patient diagnosis, treatment, and outcomes of a particular disease, condition, or exposure.^1^ Unlike electronic medical records (EMRs), which are primarily created for individual patient care, clinical registries play an important role in improving the quality of patient care, supporting research, guiding clinical practice, reducing healthcare costs, and providing information to support healthcare policy and regulation.^2–6^ By providing a comprehensive repository of patient information, clinical registries enable healthcare providers to track disease characterization,^7^ monitor treatment effectiveness,^8^^,^^9^ and identify areas for improvement in patient care.^10^^,^^11^ By leveraging EMR data, clinical registries can efficiently gather real-world clinical information without duplicating data collection efforts. Figure 1 illustrates the relationship between EMRs and clinical registries.

**Figure 2.**
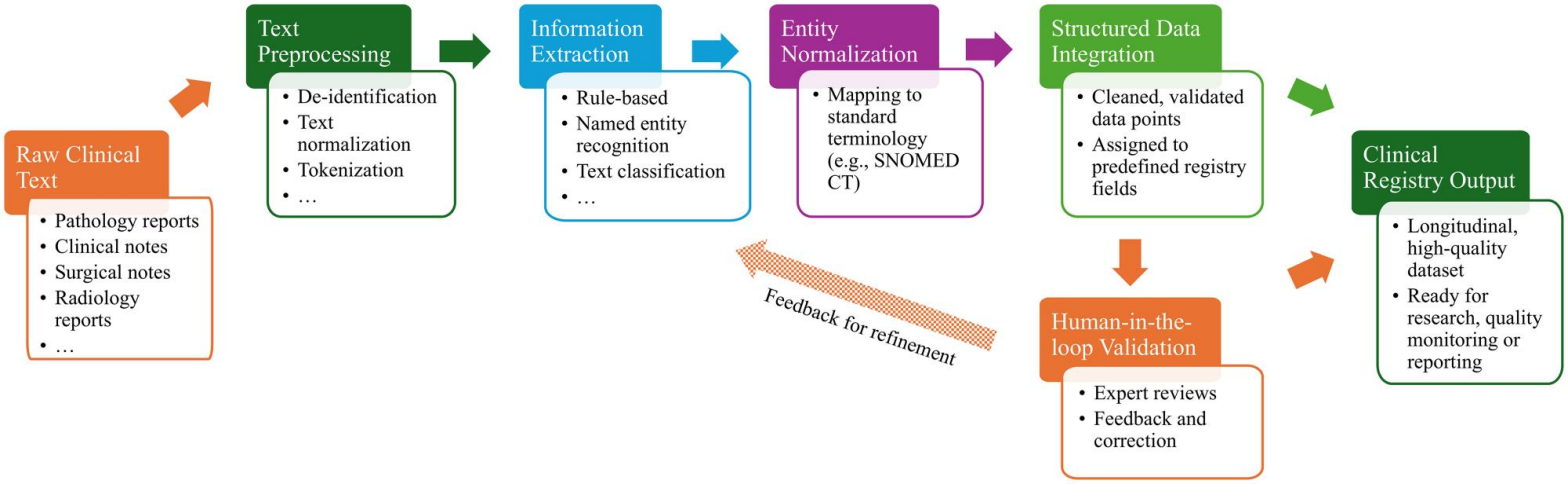
From raw clinical text to curated registry fields: a conceptual transformation pipeline with a human-in-the-loop mechanism. Expert feedback can be used to refine extraction performance iteratively.

Clinical text is ubiquitous in EMRs, as it serves as an important communication channel between healthcare providers.^12^^,^^13^ Up to 80% of the content of EMRs consists of unstructured data, including clinical text.^14^ This contains detailed and nuanced information about patient care that is often not captured in structured data.^15^ Significant patient information that could be of value for populating clinical registries, such as social determinants, symptoms, conditions, interventions, brief medical history, disease history, and medication history, remains locked within clinical text.^12^^,^^16^^,^^17^ Clinical registry population typically involves a pipeline that transforms clinical text into coded, structured variables aligned with predefined registry data elements (eg, diagnosis, procedure date, or outcome status), as illustrated in Figure 2.

Manually extracting relevant information from clinical text has been widely used to create and maintain clinical registries. However, this is expensive, labor-intensive, time- and resource-consuming, and prone to human error.^17–20^ Some studies report that the human error rate is over 28% and the inter-rater reliability is variable.^20–22^ By addressing the challenges of manual data extraction from large volumes of clinical text, natural language processing (NLP) has significant potential for constructing clinical registries. While NLP has been broadly applied in other clinical domains, such as case detection and event extraction,^23^^,^^24^ populating clinical registries presents a distinct set of informatics challenges that go beyond general information extraction. Transforming narrative text to populate registry fields typically requires processing large-scale clinical text spanning extended timeframes, extracting a wide range of heterogeneous data elements at a high level of granularity, and ensuring that information remains current through continuous updates. These demands introduce complexities related to scalability, consistency, and longitudinal integration of extracted data.

Over the past decade, NLP techniques have evolved from hand-crafted rules to the realm of large language models (LLMs). Several NLP information extraction methods have been adapted for populating clinical registries.^25–27^ However, this is challenging. Rule-based NLP methods require domain experts to manually curate comprehensive rules for capturing all possible linguistic expressions,^26^ while the effectiveness of machine learning-based NLP methods relies on the quality and quantity of annotated datasets. Clinical registries often contain dozens of data elements,^18^ making preparing sufficient annotated data a significant challenge. Recently, generative LLMs have emerged as a promising solution, providing the ability to populate clinical registries and potentially reducing the need for annotated data.^28^

Several systematic reviews have been conducted to review information extraction methods from clinical text for case detection of specific conditions,^16^^,^^29–31^ provide an overview of specific registries,^32–34^ review the impact of registries on healthcare quality,^35–37^ examine the quality of registries,^38^^,^^39^ or outline the usage of registries.^40^ However, no reviews have specifically evaluated the use and effectiveness of NLP methods in extracting information from clinical text to populate clinical registries.

In this systematic review, we aimed to identify, evaluate, and summarize the current state of NLP for populating clinical registries, that is, fully automated extraction of new information from clinical text into specified structured registry fields. Specifically, we examine (1) the characteristics of clinical registries that have applied NLP methods, (2) the NLP methods used, (3) the accuracy and limitations of these methods, and (4) their reproducibility and real-world implementation. By aligning recent NLP advances with the complex demands of registry development, this review fills a critical gap and informs future efforts toward more scalable, efficient, and accurate population of clinical registries.

## Materials and methods

In this review, we followed the reporting guidelines of the Preferred Reporting Items for Systematic Reviews and Meta-Analyses (PRISMA) 2020 statement.^41^ The main and abstract item checklists were reported in Appendix S1. The systematic review protocol was registered in PROSPERO (CRD42024536122).

### Eligibility criteria

We included studies that developed or applied NLP techniques to extract information from clinical text in EMRs for populating clinical registries and provided detailed information on model architecture and performance evaluation. Studies that did not use NLP as the main method for information extraction or solely validated existing NLP methods were excluded from this review. Additionally, we excluded studies that only used clinical registry data without focusing on populating registries. See Appendix S2 for details of the inclusion and exclusion criteria.

### Information sources and search strategy

We searched 5 databases, including Embase, PubMed, Scopus, Web of Science, and ACM Digital Library, to identify all relevant articles. We restricted the search to English-language articles published from 1^st^ January 2000 to 3^rd^ June 2025 in peer-reviewed journals or conference proceedings. The search terms were grouped into 2 distinct sets, combined using the OR operator within each set and the AND operator across sets for each query. The first set of search terms related to the clinical registry and its synonyms and abbreviations, such as “clinical registry*,” “health registry*,” “patient registry*,” and “disease registry*.” The second set corresponded to the NLP techniques used for information extraction such as “natural language processing,” “deep learning,” “machine learning,” “artificial intelligence,” “neural network,” “text mining,” “computational linguistics,” “language model,” “LLM,” “RAG,” “retrieval-augmented generation,” “transformer,” “GPT,” and “ChatGPT.” The search terms were selected to be comprehensive to maximize the coverage of the articles. We searched for the title, abstract, and keywords fields only. The queries for each database are available in Appendix S3.

### Study selection

The search results were imported into the Covidence systematic review software. Any studies retrieved from citation searching were manually added to Covidence. The duplicates were automatically removed by the software using a matching algorithm that identifies records with overlapping metadata (ie, title, year, volume, and author).^42^ Two independent reviewers evaluated the eligibility of screened studies through title and abstract review, as well as full-text review. Any conflicts were resolved through discussion between the 2 reviewers in scheduled consensus meetings, with the third senior reviewer making the final decision if necessary. During the title and abstract review, 61 out of 5673 studies required discussions. At the full text review stage, 10 out of 45 required discussions. All discrepancies were resolved through meetings between the 2 reviewers, without the need for a third reviewer’s adjudication.

### Data collection and data items

A customized data extraction form was developed based on the checklist for Critical Appraisal and Data Extraction for Systematic Reviews of Prediction Modelling Studies (CHARMS).^43^ The data fields in the form included basic study information, data source characteristics, a description of the clinical registry, data elements extracted from clinical text, NLP method definitions, NLP algorithms used, model performance and evaluation, availability of research outcomes, and implementation. See Appendix S4 for the data collection form, which was published in our Covidence project. Two reviewers independently extracted all the data fields for the included studies. Any discrepancies were resolved through inter-rater discussion in scheduled consensus meetings, with the third senior reviewer making the final decision if necessary.

To facilitate transparency and accessibility, we provided a supplementary spreadsheet (Supplementary_consolidated_study_information.xlsx) consolidating the extracted study information into a single table. This file is available as an online supplement in Excel format.

### Quality assessment

The risk of bias of the included studies was assessed using a customized tool, adapted from the Prediction Model Risk of Bias Assessment Tool (PROBAST),^44^ modified to reflect NLP-specific considerations. See Appendix S5 for details. The form utilized 12 questions to rate the risk of bias as high, low, or unsure across 4 domains: data, modeling, outcome, and reproducibility. It used the same extraction procedure as data collection.

### Research questions and data synthesis

Aligned with the aims of this review, we posed 4 main research questions and key areas for data synthesis (Box 1).

Box 1. Research questions and key areas for data synthesis.

(1) What are the characteristics of clinical registries in the included studies?

  • Summary of the patient cohort

  • Overview of clinical text characteristics

  • Description of clinical registry data elements extracted from clinical text

(2) What NLP approaches have been proposed to automate the population of clinical registries?

  • Summary of annotated data used for model training

  • Overview of the defined NLP methods

  • Summary of NLP algorithms used

(3) What are the performance and limitations of these NLP approaches?

  • Summary of evaluation methods

  • Overview of evaluation metrics and scores

(4) What is the reproducibility and implementation status of these NLP approaches in real-world settings?

  • Summary of the availability of data, source code, and trained models

  • Overview of implementation status in practice

Due to the substantial heterogeneity in clinical text sources, NLP methods, and extracted data elements across included studies, a meta-analysis was not conducted. Instead, we synthesized the findings from the included studies using a narrative synthesis approach, supported by tabulation and visualization, to identify evidence addressing the research questions.

## Results

**Figure 3.**
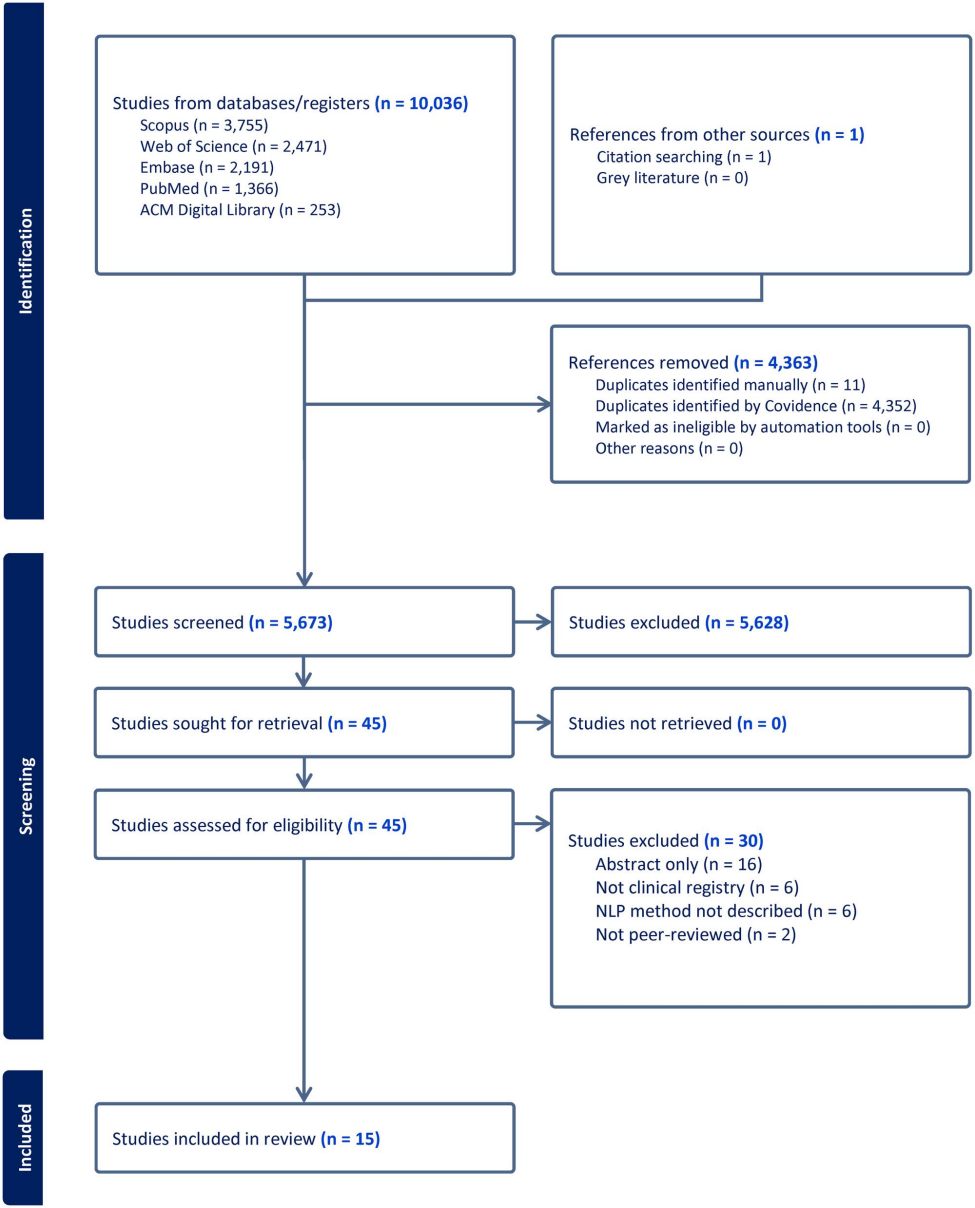
Study selection process in Covidence software.

Figure 3 illustrates the selection process for identifying eligible studies. We initially identified 10 036 studies for screening from the 5 selected databases and one from citation searching. After removing 4352 duplicates identified by Covidence and 11 duplicates identified manually, 5673 studies were screened. The title and abstract review led to the removal of 5628 studies and the retention of 45 studies for full-text review. After a full text review, we excluded 30 studies for the following reasons: (1) They were abstract only,^45–60^ (2) NLP methods were not described,^61–66^ (3) they did not populate clinical registries,^67–72^ and (4) they were not peer-reviewed.^73^^,^^74^ The remaining 15 studies were included for data extraction in the review.

### Study characteristics

Table 1 summarizes the characteristics of the included studies. These studies were published between 2010 and 2025 and conducted in 7 countries: Australia, Canada, China, Germany, the Netherlands, Taiwan, and the United States. Most studies (53%, *n *= 8) were published after 2020, and 53% (*n *= 8) were conducted in the United States. The studies covered a wide range of medical fields. The 2 main clinical areas were oncology (40%, *n *= 6), including intraductal papillary mucinous neoplasms (IPMNs), breast oncology, metastatic prostate cancer, lung cancer, and unspecified cancer, and orthopedics (13%, *n *= 2), including spine surgery and anterior cruciate ligament reconstruction operations. Other clinical areas included cardiology, neurology, gynecology, ophthalmology, nephrology, and infectious diseases. The research data used by studies spanned from 1997 to 2023 and were retrieved from retrospective cohorts such as EMRs (46%, *n *= 7), existing registries (46%, *n *= 7), and COVID-19 patient data from the National Library of Medicine (NLM) (7%, *n *= 1). Ten studies (67%) used data extracted from a single center, 4 studies (27%) used data from multiple centers, and one study did not provide this information.

**Table 1.** Study characteristics.

Study	Country	Clinical registry	Study population	Number of sites data were extracted	Data timeframe	Patient cohort
Al-Haddad et al. 2010^25^	United States	IPMNs database	Existing registry	1	January 2007-December 2007	5694 IPMNs patients
Davis et al. 2013^19^	United States	Multiple sclerosis database	Retrospective cohort	1	1997-2013	5789 multiple sclerosis patients
Rastegar-Mojarad et al. 2017^75^	United States	Gynecology surgery registry	Existing registry	1	1998-2014	7123 gynecology surgical patients
Tian et al. 2019^17^	China	Chinese coronary artery disease registry	Existing registry	15	NI	150 coronary artery disease patients
Alawad et al. 2021^26^	United States	Cancer registry	Existing registry	2	2004-2018	NI
Percha et al. 2022^18^	United States	Breast oncology registry	Retrospective cohort	1	2010-2020	7439 breast oncology patients
Cheung et al. 2023^20^	United States	Spine surgery registry	Retrospective cohort	1	2013-2023	NI
Macri et al. 2023^76^	Australia	Ophthalmic diseases registry	Retrospective cohort	1	2019-2022	NI
Barr et al. 2023^77^	Canada	Manitoba glomerular diseases registry	Retrospective cohort	1	2002-2019	2278 kidney biopsy patients
Raza et al. 2023^78^	Canada	COVID-19 disease database	National Library of Medicine	NI	March 2022—June 2022	5000 COVID-19 patients
Bosch et al. 2023^79^	The Netherlands	Metastatic prostate cancer registry	Retrospective cohort	3	2016-2021	11 494 mHSPC or CRPC patients
Tavabi et al. 2024^80^	United States	Registry of ACL reconstruction operations	Retrospective cohort	1	2000 - 2021	NI
Dai et al. 2024^81^	Taiwan	Taiwan Cancer Registry Center (TCRC)	Retrospective cohort	1	2018 - 2020	507 lung cancer patients
Lee et al. 2024^82^	United States	The Society of Thoracic Surgeons (STS) Adult Cardiac Surgery registry (STS-ACS)	Retrospective cohort	3	2011 - 2023	85 922 adult cardiac surgery patients
Mou et al. 2024^83^	Germany	Cancer registry	Retrospective cohort	1	NI	50 breast cancer patients

### Study quality assessment

**Figure 4.**
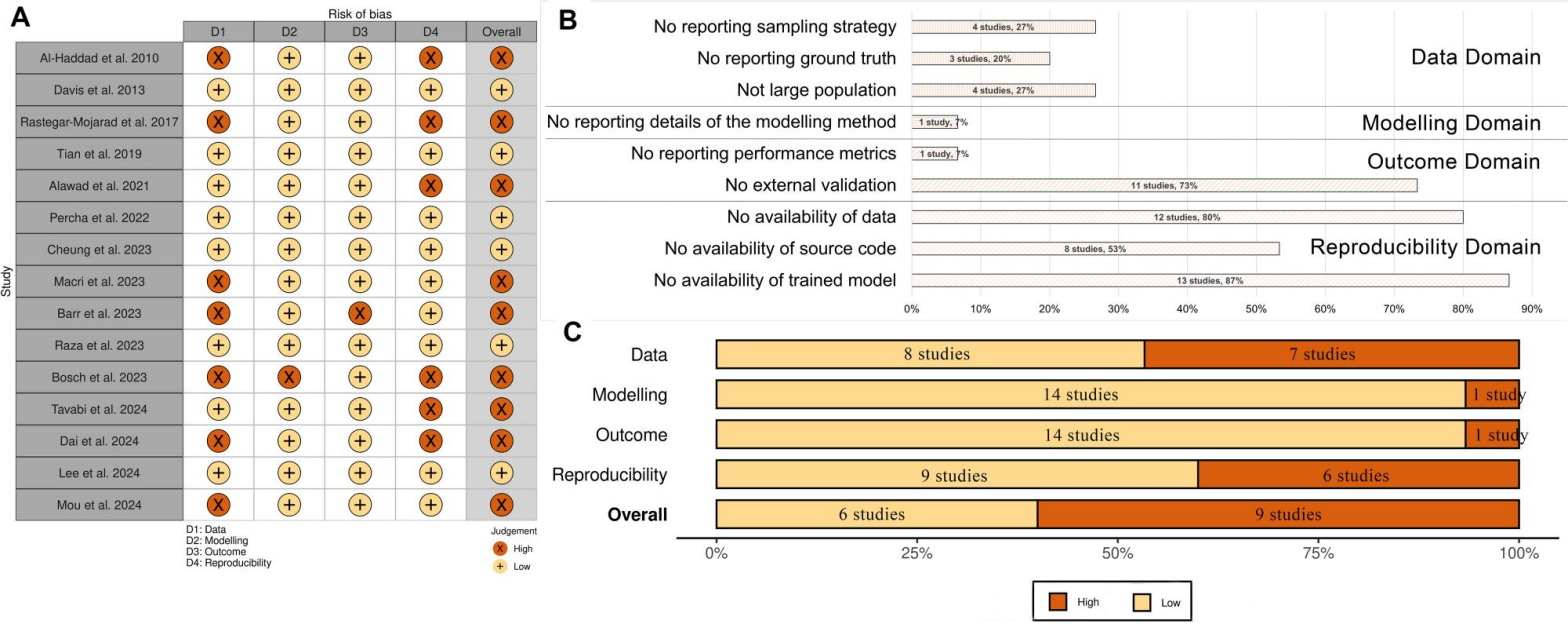
Study risk of bias assessment. (A) Domain-level judgments for each study. (B) Frequency of risk of bias concerns across studies. (C) Distribution of risk of bias judgments within each bias domain.

The study quality appraisal results are shown in Figure 4, which was created using the robvis tool.^84^ Six studies showed a low overall risk of bias. Of the other studies, one (11%) had a high risk in 3 domains, 4 (44%) had a high risk in 2 domains, and 4 (44%) had a high risk in one domain. The key issues in the reproducibility domain were no availability of data (80%, *n *= 12), no availability of source code (53%, *n *= 8), and no availability of the trained model (87%, *n *= 13), as shown in Figure 4B. In the “Data” domain, nearly half of the studies had a high risk of bias. Among these, either the sampling strategy or the ground truth dataset was not well reported. Although only one study had a high risk of bias in the “Outcome” domain, 73% (*n *= 11) of the studies did not perform external validation on their methods. Almost all studies (93%, *n *= 14) had a low risk of bias in the “Modelling” domain, except one study, which did not report the details of the modelling method. See Appendix S6 for more details.

### Clinical text data used

Table 2 illustrates the characteristics of clinical text data used in the included studies. Two of the 15 studies did not specify which types of clinical text they used. Six studies (40%) extracted clinical registry data elements from pathology reports, 4 (27%) from surgical notes, 2 (13%) from clinical notes, one (7%) from outpatient visit notes, and one (7%) from case reports. Nearly all the clinical text data were in English except for 2 studies that used Chinese data and one study that used Dutch data. Twelve studies (80%) reported the number of clinical text documents in the research data cohort. The smallest dataset was 50 documents, and the largest one was 410 064. Only 2 studies mentioned the length of the clinical text documents.

**Table 2.** Data characteristics of the included studies.

Study	Clinical text type	Clinical text language	Number of clinical text documents	Length of clinical text	Methods used for data annotation	Domain experts for annotation	Number of Annotators	Inter-rater agreement (Kappa)
Al-Haddad et al. 2010^25^	Pathology reports	English	165 000	NI	Manual annotation	Yes	NI	NI
Davis et al. 2013^19^	Clinical notes	English	5849	NI	Manual annotation	Yes	3	0.96
Rastegar-Mojarad et al. 2017^75^	Surgical notes	English	7123	NI	Existing annotated data	No	NA	NA
Tian et al. 2019^17^	NI	Chinese	NI	NI	Manual annotation	Yes	3	NI
Alawad et al. 2021^26^	Pathology reports	English	410 064	NI	Manual annotation	Yes	NI	NI
Percha et al. 2022^18^	Clinical notes Laboratory notes Pathology reports	English	NI	An average of 374 sentences	Automated annotation	No	NA	NA
Cheung et al. 2023^20^	Surgical notes	English	31 502	NI	Manual annotation	Yes	3	NI
Macri et al. 2023^76^	Outpatient visit notes	English	33 455	NI	Manual annotation	Yes	1	NA
Barr et al. 2023^77^	Pathology reports	English	2421	NI	Manual annotation	Yes	1	NA
Raza et al. 2023^78^	Case reports	English	5000	NI	Manual annotation	Yes	4	0.75
Bosch et al. 2023^79^	NI	Dutch	NI	NI	Manual annotation	Yes	NI	NI
Tavabi et al. 2024^80^	Surgical notes	English	15 277	NI	Manual annotation	Yes	3	NI
Dai et al. 2024^81^	Pathology and imaging reports	Chinese	7378	NI	Manual annotation	Unspecified	4	0.84
Lee et al. 2024^82^	Operative reports	English	85 922	An average of 170 words	Existing annotated data	No	NI	NI
Mou et al. 2024^83^	Pathology reports	German	50	NI	Manual annotation	Yes	NI	NI

Abbreviations: NI, no information; NA, not applicable.

### Clinical registry data elements extracted

**Figure 5.**
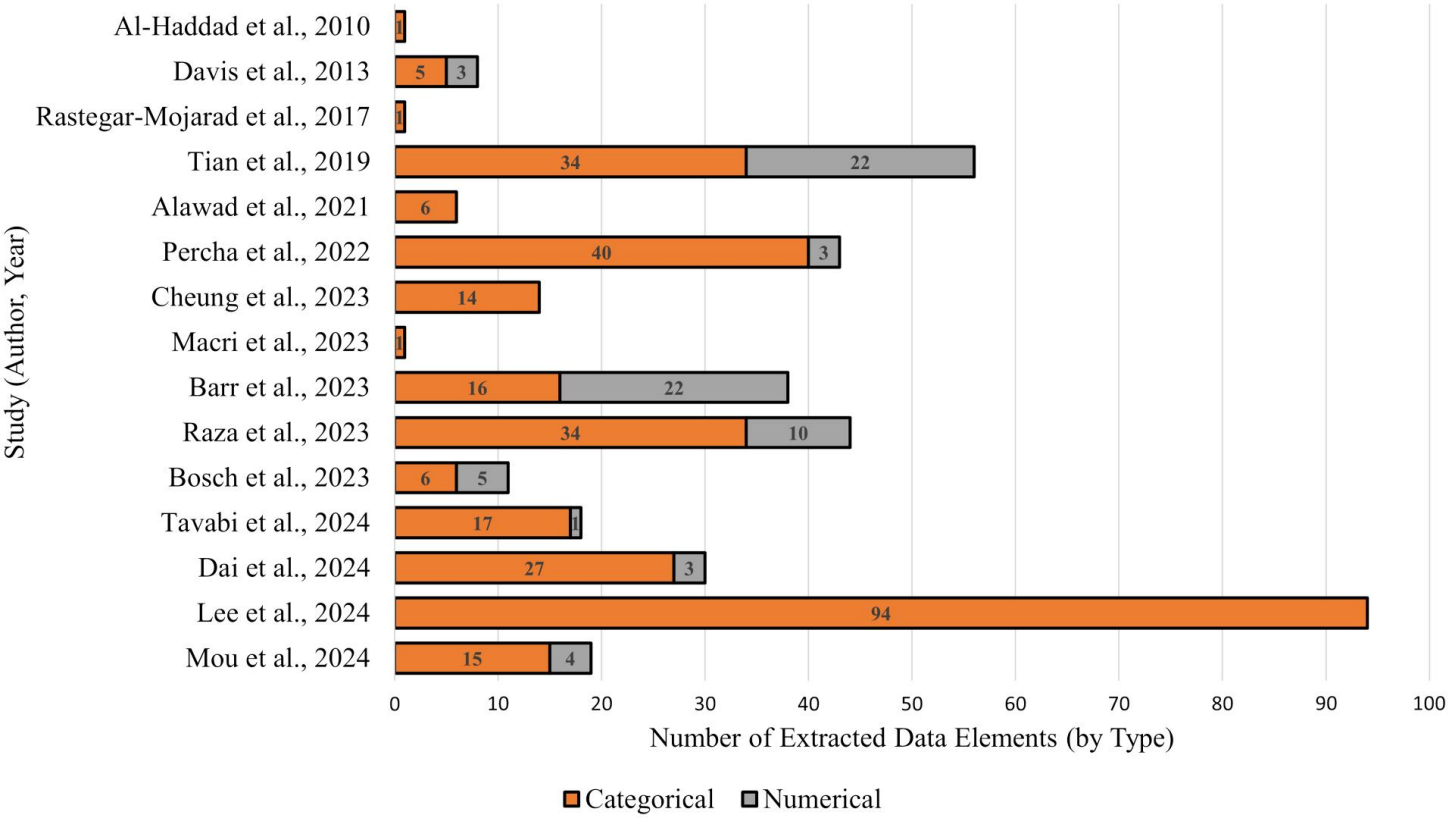
Number of clinical registry data elements extracted from clinical text in each study, categorized by data type (orange bars represent categorical elements; gray bars represent numerical elements).

As shown in Figure 5, the majority of extracted data elements across studies are categorical, with only one study extracting more numerical elements than categorical ones. Various data elements were extracted in different studies. For example, Tian et al.^17^ extracted 56 data elements, primarily focused on patient demographics, disease history, family members, laboratory results, and image examination results. Raza et al.^78^ extracted 44 data elements, including clinical factors and social determinants of health associated with infectious diseases. Tavabi et al.^80^ extracted 18 data elements, which contain surgical procedures and devices and anatomical structures and injuries. Three studies only extracted one data element related to diagnosis and procedure.^25^^,^^75^^,^^76^ In terms of data types, the number of categorical data elements varied across different studies, with the majority having a significant proportion of categorical data compared to numerical data. The lists of data elements of all the studies are shown in Appendix S7.

### NLP approaches

As shown in Table 3, the NLP methods of the included studies fell into 6 categories: rule-based (42%, *n *= 5), text classification (27%, *n *= 4), named entity recognition (NER) (20%, *n *= 3), natural language inference (NLI) (7%, *n *= 1), text mining (7%, *n *= 1), and generative LLMs (7%, *n *= 1).

**Table 3.** Natural language processing (NLP) approaches proposed by each study for populating clinical registries.

Study	NLP Method	Model algorithms	Annotated Corpus Size & Configuration	Internal validation	External validation	**Performance (%)** ^b^
Al-Haddad et al. 2010^25^	Rule-based	Regular expression and additional algorithmic rules	NI	Non-random split	ND	Precision: 95.5 Sensitivity/recall: 97.5
Davis et al. 2013^19^	Rule-based	Regular expression	Training: 899 Test: 4890	Random split	ND	Macro F1: 74.5 (49.0-94.0)^a^ Macro precision: 92.1 (87.0-99.0)^a^ Macro sensitivity/recall: 65.8 (33.0-98.0)^a^ Macro specificity: 95.5 (81.0-100.0)^a^
Rastegar-Mojarad et al. 2017^75^	Text classification	Naive Bayes, random forest, SVM	NI	Random split Cross-validation	ND	F1: 75.0-86.4 Precision: 64.1-80.2 Sensitivity/recall: 73.3-94.6
Tian et al. 2019^17^	Rule-based	Regular expression	NI	Random split	ND	Accuracy: 93.29
Alawad et al. 2021^26^	Text classification	MT-CNN	Training: 262 561 Validation: 65 626 Test: 81 877	Random split	ND	Macro F1: 58.5 Micro F1: 82.7
Percha et al. 2022^18^	Natural language inference	Pretrained NLI language model	NA	No split^c^	ND	Accuracy: 51.2
Cheung et al. 2023^20^	Rule-based	Regular expression	Training: 100 Test: 100	Random split	ND	F1: 81.0 Precision: 91.9 Sensitivity/recall: 72.3 NPV: 99.1 Accuracy: 98.9 Specificity: 99.8
Macri et al. 2023^76^	NER	Spacy NER model	Training: 1538 Test: 385	Random split	ND	F1: 81.3 Precision: 81.6 Sensitivity/recall: 81.0
Barr et al. 2023^77^	Rule-based	Regular expression	Training: 80 Test: 2511	Random split	ND	NI
Raza et al. 2023^78^	NER	Transformer-BiLSTM-CRF	Training: 3500 Test: 1500	Random split Cross-validation	Benchmark datasets	F1: 94.9
Bosch et al. 2023^79^	Text mining	Off-the-shelf NLP software (CTcue)	NA	Random split	ND	Accuracy: 87.4
Tavabi et al. 2024^80^	Text classification	SE-K, SE-E, BERT, and Ensemble model of these	Training: 800 Test: 200	Random split Cross-validation	Temporal	Sensitivity/recall: 97.9 (92.0-100.0)^a^ Specificity: 97.1 (83.0-100.0)^a^ AUC: 97.6 (91.0-100.0)^a^ Accuracy: 98.7 (95.0-100.0)^a^
Dai et al. 2024^81^	NER	Rule-based and RoBERTa-BiLSTM-CRF	Training: 5902 Test: 1476	Random split	Temporal	Macro F1: 91.8 (85.6-100.0)^a^ Macro Precision: 92.5 (86.3-100.0)^a^ Macro Sensitivity/recall: 91.1 (84.8-100.0)^a^
Lee et al. 2024^82^	Text classification	BioClinicalBERT	NI	Stratified by site, registry version and procedure	Temporal and geographic	F1: 45.5 Precision: 46.2 Sensitivity/recall: 47.0
Mou et al. 2024^83^	Generative LLMs	Mixtral-8x7B and GPT-4	Training: 25 Test: 25	Random split	ND	Correctness: 95.0 Completeness: 97.0

Abbreviations: AUC, the area under the Receiver Operating Characteristic (ROC) curve; BERT, Bidirectional Encoder Representations from Transformers; BiLSTM-CRF, bi-directional long short-term memory-conditional random field; LLMs, large language models; MT-CNN, multitask convolutional neural network; NA, not applicable; ND, not done; NER, Named entity recognition; NI, no information; NLI, natural language inference; NLP, natural language processing; NPV, negative predictive value; SE-E, sentence extractor with embeddings; SE-K, sentence extractor with keywords; SVM, support vector machine.

a The study only reported the results for each data element. The macro-average results were calculated in this review.

b This column lists the results as provided by each study. For studies that included multiple methods, we reported the range of performance scores. For the studies that did not report an overall performance, the macro-average value across the extracted data elements was calculated and the range for each metric was also provided. The most commonly used metric (F1) is presented first. All metrics are micro-averaged unless explicitly marked as macro.

c This study adopted a pretrained NLI model trained on other datasets to evaluate its performance on their corpus. All data used in the study comprised the test set, so no additional data splitting was required.

For rule-based tasks, regular expressions (regex) were the main technique used to match and extract specific information from clinical text, based on manually defined patterns and keywords, often guided by domain knowledge bases.^17^^,^^19^^,^^20^^,^^25^^,^^77^

Text classification approaches assign predefined data elements to the given clinical text. The studies employed shallow classifiers for procedure extraction,^75^ multi-task deep learning classification models for cancer characteristics,^26^ ensemble models with a majority mechanism for anterior cruciate ligament (ACL) details,^80^ and fine-tuned pretrained LLMs for adult cardiac surgery registry procedural elements.^82^

Named entity recognition involves identifying and classifying named entities in text into predefined categories and can be used to extract many entities. Various methods have been used by the included studies to extract clinical registry data elements, such as training a bespoke spaCy NER model to extract diagnosis information for an ophthalmic disease registry,^76^ a transformer-based model for populating a COVID-19 disease registry,^78^ and a hybrid neural symbolic system with weighted rules for lung cancer registry coding.^81^

Natural Language Inference (NLI), also known as textual entailment, determines the relationship between a premise and a hypothesis, which makes it suitable for information extraction applications. One study^18^ applied several pretrained NLI models to clinical, laboratory, and pathology notes to infer information about 43 different breast oncology registry fields.

Bosch et al.^79^ embedded an existing text mining tool (CTcue)^85^ into the NLP pipeline to replace manual data collection for populating disease-specific patient registries. The tool used a hybrid method that combined rules and machine learning algorithms to extract information from text.

Generative LLMs were explored for converting pathology reports to a structured data model using tailored prompts, enabling automatic population of the oncology registry. To improve performance, multiple prompting strategies, such as zero-shot, few-shot, and chain-of-thought methods were evaluated.

### Validation methods and evaluation metrics

Eleven of the included studies (73%) relied only on left-out test sets for internal validation, with no external validation. The evaluation metrics used in the studies were diverse. Recall (sensitivity) and F1 were the most common metrics, used by 8 studies (53%), followed by precision (positive predictive value) (40%, *n *= 6), accuracy (33%, *n *= 5), specificity (20%, *n *= 3), AUC (the area under the ROC curve) (7%, *n *= 1), and NPV (negative predictive value) (7%, *n *= 1). For generative LLMs, correctness and completeness were used to assess the quality of extracted outputs.

### NLP performance

Across studies, performance varied by NLP method, clinical domain, and text type. Rule-based methods achieved high performance in semi-structured text, particularly surgical notes in a spine surgery registry^20^ (Accuracy: 98.9%). In contrast, studies applying machine learning classifiers to heterogeneous clinical notes showed more variable results, with micro F1-scores ranging from 45.5% to 94.9% and recall from 58.5% to 91.8%. A recent generative LLM approach^83^ achieved high accuracy in populating a cancer registry. See Table 3 for more details on model performance.

### Reproducibility and implementation

We summarized the availability of research data, method source code, and trained models or rules to evaluate if the proposed NLP approaches could be reproduced or applied to other datasets for populating clinical registries. We also collected information about the implementation of the NLP approaches in real-world clinical practice settings. See Appendix S8 for details. Only one study shared its data, which was COVID-19 data extracted from the NLM. Seven studies (47%) made their source code accessible, and 2 studies (13%) released the rules or trained models. Five studies (33%) reported implementation of their proposed NLP approaches to populate real-world clinical registries: the Chinese Coronary Artery Disease Registry,^17^ the Manitoba Glomerular Diseases Registry,^77^ a metastatic prostate cancer registry (CAPRI-3),^79^ a registry of anterior cruciate ligament surgeries,^80^ and the Taiwan Cancer Registry.^81^

## Discussion

This review is the first to evaluate the use and effectiveness of NLP methods for populating clinical registries from clinical text. By examining the characteristics of clinical registries using NLP methods, detailing the methods employed, assessing their performance, and exploring their practical implementation, this review offers a comprehensive overview of NLP’s role in the development of clinical registries. It provides valuable insights to guide future research and inform practical applications in real-world settings.

Only 15 studies published since 2010 met our inclusion criteria, highlighting the limited application of NLP methods for populating clinical registries to date. However, this review identified a growing trend in the use of NLP methods for this task, particularly after 2020. This surge coincides with not only the broader adoption of EMRs,^86^ increased availability of clinical text, and heightened interest and investment in AI-driven health research,^87^ but also advancements in NLP techniques, driven by the success of foundational LLMs such as BERT^88^ and the emergence of generative LLMs such as ChatGPT.^89^ While only one of the 15 included studies used generative LLMs, 3 (20%) employed foundational LLMs (eg, BERT, BioClinicalBERT) to extract clinical registry data elements from various clinical reports and notes.

This review highlights several methodological weaknesses that hinder the adaptation and effectiveness of NLP methods for populating clinical registries. Rule-based methods use regular expressions to match specific patterns or keywords for data element extraction from clinical text. However, this approach requires domain experts to manually curate a comprehensive set of rules according to the content and structure of clinical text. This is not only time-consuming and labor-intensive but also lacks adaptability. Moreover, rule-based approaches can achieve a much lower recall compared to a relatively high precision,^19^^,^^20^ which means that they might miss relevant information that does not match the predefined rules.

Text classification uses various feature extraction methods, such as n-grams, TF-IDF, doc2vec, BERT, and BioClinicalBERT, and different classifiers to categorize clinical text into data elements. Two studies^75^^,^^80^ leveraged binary classifiers to extract data elements separately. However, training one model per data element was time- and resource-consuming, making it unsuitable for extracting a large number of data elements. In contrast, Alawad et al. utilized a multi-task multi-class classifier to simultaneously extract 6 cancer characteristics from cancer pathology reports.^26^ Nonetheless, text classification methods are not appropriate for extracting non-categorical data elements such as age, weight, admission date, and dosage.

Named entity recognition is a token-level classification task that aims to identify word spans for predefined entity types, but it does not inherently link these spans to standard classes or ontologies. Entity normalization is a crucial downstream step for achieving semantic interoperability in clinical registries. For example, in the case of tumor anatomic location sites, additional effort is required to code the extracted entities into 70 sites for secondary research use.^26^ This finding was also reported for an ophthalmic registry.^76^

The studies that used rules-based, text classification, or NER methods (12 studies in total, 80%) all relied heavily on manually annotated datasets. Studies using rule-based methods typically used hundreds of annotated clinical text documents, while the studies using supervised machine learning used thousands of annotated documents. The quality of labeled data determines the accuracy and quality of supervised machine learning models.^90^ To get high-quality, well-labeled data, experienced domain experts need to follow a standard procedure for annotation. For instance, at least 2 independent raters should annotate the data using predefined guidelines, with a consensus process to resolve disagreements through discussion or involvement of a third rater if necessary. Consequently, the methods used in most of the included studies may not substantially reduce costs or labor requirements, particularly given the likely need for periodic updates and retraining of NLP models to maintain accuracy and relevance.

Unlike text classification and NER methods, NLI can use pretrained language models to determine the relationship between a premise (clinical text) and a hypothesis (data element). These models have been trained on some publicly available annotated NLI datasets (eg, SNLI,^91^ MNLI,^92^ FEVER-NLI,^93^ and ANLI^94^) based on foundation language models such as ALBERT, RoBERTa, and XLNet. One included study used several pretrained NLI models to extract relevant information from clinical text without any further training.^18^

One further study integrated an off-the-shelf text mining tool into the information extraction pipeline.^79^ This achieved a much lower accuracy score than other studies that used supervised machine learning methods, and the performance of the pipeline was largely dependent on the text mining tool itself.

Generative LLMs, trained on vast amounts of text data, have demonstrated exceptional performance and remarkable capabilities across various tasks.^95^ As a result, an increasing number of studies in other domains are integrating generative LLMs for information extraction tasks based on a generative paradigm.^96^ Although only one of the included studies used generative LLMs, this may be a promising direction for future research. Techniques such as prompt tuning and retrieval-augmented generation (RAG) offer practical pathways for adapting LLMs to populate clinical registries, noting, however, the resource-intensive nature of generative LLMs and the potential challenges related to data protection and privacy.

The studies included used diverse clinical text data to extract a wide range of data elements. This highlights the complexity of automating clinical registry construction. Most of the proposed methods in the studies, designed for specific clinical texts and registries, were difficult to adapt to different scenarios. For example, the rules designed for extracting IPMN^25^ from pathology reports could not be applied to identify procedural details in surgical reports.^75^ The machine learning model^26^ trained for identifying tumor information from pathology reports cannot be used to extract patient demographic information from case reports. Similar challenges were noted in other studies.^78^^,^^80^ This highlights the need for more flexible and generalizable methods to support broader applications in clinical registry construction.

Among the included studies, the median micro F1-score was 81.2% with a range of 45.5%-94.9%. Of the 5 studies that reported accuracy, 4 exceeded 85%, aligning with human abstraction accuracy, which typically ranges from 72% to 95% depending on the task.^20–22^ These findings highlight both the promise and the limitations of NLP methods: while some approaches achieve near-human-level performance in specific domains, overall effectiveness remains variable, indicating that most systems remain experimental rather than ready for routine use in clinical registry settings.

This review also identified several additional limitations of the included studies that impact their broader applicability and reproducibility. A key concern is the lack of external validation in most studies, raising questions about their robustness, trustworthiness, and generalizability to different clinical registry settings. Without evaluation on independent datasets, performance estimates may be optimistic and unreliable. Current best practices recommend external validation using temporal or geographic data to assess robustness. Furthermore, the unavailability of data, source code, and models restricts other researchers from replicating the studies or adapting the methods to their specific context. Additionally, descriptions of real-world implementations of NLP methods in registries were confined to the current studies, with no proposals addressing continuing improvement and maintenance.

Future research should focus on developing adaptable NLP frameworks that are portable across clinical registries. This may include the use of transfer learning with generative LLMs to enable efficient adaptation with minimal annotation, integration of active learning to direct low-confidence extractions for human review, and periodic re-validation as new data accrue, alongside standardized definitions of registry data elements to improve reproducibility and cross-registry generalizability.

### Limitations

There are some limitations to this systematic review. First, we may have overlooked studies published in languages other than English and the NLP methods proposed in gray literature, and papers only presented in certain specialized computer science venues (eg, IEEE Xplore). This may have led to the omission of relevant NLP efforts that could offer additional insights into novel approaches and completeness of our conclusions. Second, we did not perform a meta-analysis of NLP method performance due to the significant heterogeneity of research data sources and extracted data elements. Third, we may have neglected some potentially relevant NLP methods by excluding studies using clinical text that did not focus on populating clinical registries but may have created a clinical registry after publication of their clinical NLP methods, as well as studies conducted in the general domain.

## Conclusion

This review highlights the growing trend of using NLP methods to populate clinical registries from clinical text. The existing studies and NLP methods have not adequately addressed the complexity of the extracted data elements or the nuanced structure of clinical text. A lot of manual work is still required to prepare rules, label training data, or generate NLI hypotheses. Methodological flaws were identified, including the limited adaptability of rule-based approaches and the inefficiency of training one model per data element in text classification. The alternative methods, NER and NLI, also have limitations, such as the need for additional coding of extracted entities and lower performance. Additionally, the lack of external validation and the unavailability of research artifacts in many studies raise concerns about the generalizability and reproducibility of the NLP methods.

This review underscores the need for a robust and adaptable NLP framework that can handle the complexity and diverse characteristics of clinical text and data elements. While integrating generative LLMs for information extraction may potentially provide direction for future research, such directions should be pursued with careful consideration of current challenges, including accuracy, computational cost, and data privacy, as well as the limited direct evidence available now.

## Supplementary Material

ocaf176_Supplementary_Data

## Data Availability

All data extracted in this review are provided in the supplementary spreadsheet (Supplementary data exported from Covidence).
