# Supplementary material for: Using natural language processing to extract information from clinical text in electronic medical records for populating clinical registries: a systematic review
Source: J Am Med Inform Assoc. 2025 Oct 15;33(2):484–99. doi: 10.1093/jamia/ocaf176 (PMC12844598; doi:10.1093/jamia/ocaf176)
Supplement: ocaf176_Supplementary_Data [file ocaf176_supplementary_data.zip › Supplementary materials_clean.docx]

**Appendix 1: PRISMA 2020 item checklist**

| **Section and Topic** | **Item #** | **Checklist item** | **Location where item is reported** |
| --- | --- | --- | --- |
| **TITLE** | | |  |
| Title | 1 | Identify the report as a systematic review. | 1 |
| **ABSTRACT** | | |  |
| Abstract | 2 | See the PRISMA 2020 for Abstracts checklist. | 2 |
| **INTRODUCTION** | | |  |
| Rationale | 3 | Describe the rationale for the review in the context of existing knowledge. | 4-5 |
| Objectives | 4 | Provide an explicit statement of the objective(s) or question(s) the review addresses. | 5 |
| **METHODS** | | |  |
| Eligibility criteria | 5 | Specify the inclusion and exclusion criteria for the review and how studies were grouped for the syntheses. | 5 |
| Information sources | 6 | Specify all databases, registers, websites, organisations, reference lists and other sources searched or consulted to identify studies. Specify the date when each source was last searched or consulted. | 5 |
| Search strategy | 7 | Present the full search strategies for all databases, registers and websites, including any filters and limits used. | 5 |
| Selection process | 8 | Specify the methods used to decide whether a study met the inclusion criteria of the review, including how many reviewers screened each record and each report retrieved, whether they worked independently, and if applicable, details of automation tools used in the process. | 5 |
| Data collection process | 9 | Specify the methods used to collect data from reports, including how many reviewers collected data from each report, whether they worked independently, any processes for obtaining or confirming data from study investigators, and if applicable, details of automation tools used in the process. | 6 |
| Data items | 10a | List and define all outcomes for which data were sought. Specify whether all results that were compatible with each outcome domain in each study were sought (e.g. for all measures, time points, analyses), and if not, the methods used to decide which results to collect. | 6, Appendix 4 |
|  | 10b | List and define all other variables for which data were sought (e.g. participant and intervention characteristics, funding sources). Describe any assumptions made about any missing or unclear information. | 6 |
| Study risk of bias assessment | 11 | Specify the methods used to assess risk of bias in the included studies, including details of the tool(s) used, how many reviewers assessed each study and whether they worked independently, and if applicable, details of automation tools used in the process. | 6 |
| Effect measures | 12 | Specify for each outcome the effect measure(s) (e.g. risk ratio, mean difference) used in the synthesis or presentation of results. | NA |
| Synthesis methods | 13a | Describe the processes used to decide which studies were eligible for each synthesis (e.g. tabulating the study intervention characteristics and comparing against the planned groups for each synthesis (item #5)). | 6 |
|  | 13b | Describe any methods required to prepare the data for presentation or synthesis, such as handling of missing summary statistics, or data conversions. | 6 |
|  | 13c | Describe any methods used to tabulate or visually display results of individual studies and syntheses. | 6 |
|  | 13d | Describe any methods used to synthesize results and provide a rationale for the choice(s). If meta-analysis was performed, describe the model(s), method(s) to identify the presence and extent of statistical heterogeneity, and software package(s) used. | 6 |
|  | 13e | Describe any methods used to explore possible causes of heterogeneity among study results (e.g. subgroup analysis, meta-regression). | NA |
|  | 13f | Describe any sensitivity analyses conducted to assess robustness of the synthesized results. | NA |
| Reporting bias assessment | 14 | Describe any methods used to assess risk of bias due to missing results in a synthesis (arising from reporting biases). | NA |
| Certainty assessment | 15 | Describe any methods used to assess certainty (or confidence) in the body of evidence for an outcome. | NA |
| **RESULTS** | | |  |
| Study selection | 16a | Describe the results of the search and selection process, from the number of records identified in the search to the number of studies included in the review, ideally using a flow diagram. | 7-8 |
|  | 16b | Cite studies that might appear to meet the inclusion criteria, but which were excluded, and explain why they were excluded. | 7-8 |
| Study characteristics | 17 | Cite each included study and present its characteristics. | 8-10 |
| Risk of bias in studies | 18 | Present assessments of risk of bias for each included study. | 11 |
| Results of individual studies | 19 | For all outcomes, present, for each study: (a) summary statistics for each group (where appropriate) and (b) an effect estimate and its precision (e.g. confidence/credible interval), ideally using structured tables or plots. | 8-17 |
| Results of syntheses | 20a | For each synthesis, briefly summarise the characteristics and risk of bias among contributing studies. | 8-17 |
|  | 20b | Present results of all statistical syntheses conducted. If meta-analysis was done, present for each the summary estimate and its precision (e.g. confidence/credible interval) and measures of statistical heterogeneity. If comparing groups, describe the direction of the effect. | 8-17 |
|  | 20c | Present results of all investigations of possible causes of heterogeneity among study results. | NA |
|  | 20d | Present results of all sensitivity analyses conducted to assess the robustness of the synthesized results. | NA |
| Reporting biases | 21 | Present assessments of risk of bias due to missing results (arising from reporting biases) for each synthesis assessed. | NA |
| Certainty of evidence | 22 | Present assessments of certainty (or confidence) in the body of evidence for each outcome assessed. | NA |
| **DISCUSSION** | | |  |
| Discussion | 23a | Provide a general interpretation of the results in the context of other evidence. | 18 |
|  | 23b | Discuss any limitations of the evidence included in the review. | 18-20 |
|  | 23c | Discuss any limitations of the review processes used. | 20 |
|  | 23d | Discuss implications of the results for practice, policy, and future research. | 18-20 |
| **OTHER INFORMATION** | | |  |
| Registration and protocol | 24a | Provide registration information for the review, including register name and registration number, or state that the review was not registered. | 5 |
|  | 24b | Indicate where the review protocol can be accessed, or state that a protocol was not prepared. | 5 |
|  | 24c | Describe and explain any amendments to information provided at registration or in the protocol. | NA |
| Support | 25 | Describe sources of financial or non-financial support for the review, and the role of the funders or sponsors in the review. | 21 |
| Competing interests | 26 | Declare any competing interests of review authors. | 21 |
| Availability of data, code and other materials | 27 | Report which of the following are publicly available and where they can be found: template data collection forms; data extracted from included studies; data used for all analyses; analytic code; any other materials used in the review. | 21 |

**Appendix 2: Eligibility Criteria**

| **Inclusion Criteria** | **Exclusion Criteria** |
| --- | --- |
| 1. Population: any patients 2. Outcome: building clinical registries 3. Data modality: unstructured health data (clinical text) 4. Data analysis: NLP methods, pipelines or workflows 5. Study characteristics: 6. Employ NLP to extract information for clinical registries 7. Published after the year 2000 8. Language and publication status: 9. English 10. Journal articles or peer-reviewed conference proceedings only | 1. Studies focusing on non-registry datasets 2. Studies focusing on the clinical registries that did not extract any information from clinical text 3. Studies not using NLP as the main method for IE 4. Systematic or literature review |

**Appendix 3: Search queries in Embase, PubMed, Scopus, and Web of Science**

Embase (1647 results): Final search conducted on 3^rd^ June 2025.

| # | Query | Results |
| --- | --- | --- |
| 1 | (("clinical" or "health" or "patient" or "disease") adj2 ("registr*" or "repositor*" or "database*")).ab,ti. | 126,663 |
| 2 | ("natural language processing" or "NLP" or "machine learning" or "artificial intelligence" or "deep learning" or "neural network*" or "text mining" or "computational learning" or "computational linguistics" or "language model" or "LLM" or "RAG" or "retrieval-augmented generation" or "transformer" or "GPT" or "ChatGPT").ab,ti. | 405,756 |
| 3 | 1 and 2 | 2,244 |
| 4 | Limit 3 to (english language and yr="2000 -Current") | 2,191 |

PubMed (1052 results): Final search conducted on 3^rd^ June 2025.

| # | Query | Results |
| --- | --- | --- |
| 1 | "clinical registry"[Title/Abstract:~1] OR "clinical registries"[Title/Abstract:~1] OR "clinical repository"[Title/Abstract:~1] OR "clinical repositories"[Title/Abstract:~1] OR "clinical database"[Title/Abstract:~1] OR "clinical databases"[Title/Abstract:~1] OR "health registry"[Title/Abstract:~1] OR "health registries"[Title/Abstract:~1] OR "health repository"[Title/Abstract:~1] OR "health repositories"[Title/Abstract:~1] OR "health database"[Title/Abstract:~1] OR "health databases"[Title/Abstract:~1] OR "patient registry"[Title/Abstract:~1] OR "patient registries"[Title/Abstract:~1] OR "patient repository"[Title/Abstract:~1] OR "patient repositories"[Title/Abstract:~1] OR "patient database"[Title/Abstract:~1] OR "patient databases"[Title/Abstract:~1] OR "disease registry"[Title/Abstract:~1] OR "disease registries"[Title/Abstract:~1] OR "disease repository"[Title/Abstract:~1] OR "disease repositories"[Title/Abstract:~1] OR "disease database"[Title/Abstract:~1] OR "disease databases"[Title/Abstract:~1] | 668,691 |
| 2 | "natural language processing"[Title/Abstract] OR "NLP"[Title/Abstract] OR "machine learning"[Title/Abstract] OR "artificial intelligence"[Title/Abstract] OR "deep learning"[Title/Abstract] OR "neural network*"[Title/Abstract] OR "text mining"[Title/Abstract] OR "computational learning"[Title/Abstract] OR "computational linguistics"[Title/Abstract] OR "language model"[Title/Abstract] OR "LLM"[Title/Abstract] OR "RAG"[Title/Abstract] OR "retrieval-augmented generation"[Title/Abstract] OR "transformer"[Title/Abstract] OR "GPT"[Title/Abstract] OR "ChatGPT"[Title/Abstract] | 368,036 |
| 3 | 1 and 2 | 1,383 |
| 4 | Limit 3 to English, from 2000 - 2025 | 1,366 |

Scopus (2861 results): Final search conducted on 3^rd^ June 2025.

| # | Query | Results |
| --- | --- | --- |
| 1 | ( "clinical" OR "health" OR "patient" OR "disease" ) PRE/2 ( "registr*" OR "repositor*" OR "database*" ) | 137,325 |
| 2 | "natural language processing" OR "nlp" OR "machine learning" OR "artificial intelligence" OR "deep learning" OR "neural network*" OR "text mining" OR "computational learning" OR "computational linguistics" or "language model" or "LLM" or "RAG" or "retrieval-augmented generation" or "transformer" or "GPT" or "ChatGPT" | 2,871,969 |
| 3 | 1 and 2 | 3,859 |
| 4 | Limit 3 to PUBYEAR > 1999 AND PUBYEAR < 2026 AND ( LIMIT-TO ( LANGUAGE , "English" ) ) | 3,755 |

Web of Science (1828 results): Final search conducted on 3^rd^ June 2025.

| # | Query | Results |
| --- | --- | --- |
| 1 | ("clinical" or "health" or "patient" or "disease") NEAR/2 ("registr*" or "repositor*" or "database*") (Topic) | 98,237 |
| 2 | "natural language processing" or "NLP" or "machine learning" or "artificial intelligence" or "deep learning" or "neural network*" or "text mining" or "computational learning" or "computational linguistics" or "language model" or "LLM" or "RAG" or "retrieval-augmented generation" or "transformer" or "GPT" or "ChatGPT" (Topic) | 1,669,913 |
| 3 | 1 and 2 | 2,503 |
| 4 | Limit 3 to English, from 2000-current | 2,471 |

ACM Digital Library ( results): Final search conducted on 3^rd^ June 2025.

| # | Query | Results |
| --- | --- | --- |
| 1 | "clinical registr*" OR "clinical repositor*" OR "clinical database*" OR "health registr*" OR "health repositor*" OR "health database*" OR "patient registr*" OR "patient repositor*" OR "patient database*" OR "disease registr*" OR "disease repositor*" OR "disease database*" | 423 |
| 2 | "natural language processing" OR "NLP" OR "machine learning" OR "artificial intelligence" OR "deep learning" OR "neural network*" OR "text mining" OR "computational learning" OR "computational linguistics" OR "language model" OR "LLM" OR "RAG" OR "retrieval-augmented generation" OR "transformer" OR "GPT" OR "ChatGPT" | 300,641 |
| 3 | 1 and 2 | 274 |
| 4 | [E-Publication Date: (01/01/2000 TO 31/12/2025)] | 253 |

**Appendix 4: Data collection form**

*(All questions are text fields unless specified)*

0. Study information

0.1 Author

0.2 Publication year

0.3 Publication title

0.4 Publication journal

0.5 Model name

1. Research data

1.1 Source of data

1.1.1 Source of data (**Options**: Prospective cohort/Retrospective cohort/Case-control/Nested case-control/Existing registry/Randomized trial/Combined data/Aggregated data/Unclear/No information/Other)

1.1.2 Number of sites/hospitals data were extracted

1.1.3 Date of data

1.1.4 Study site (e.g., country, city)

1.2 Dataset size

1.2.1 Patient Number

1.2.2 The number of clinical notes (**Table**: training, development, and test)

1.2.3 The length of clinical notes

1.3 Clinical note types (list all the clinical notes mentioned in the study)

1.4 Language of clinical notes (**Options**: English/Spanish/Chinese/Other)

1.5 Methods used for annotating data (**Options**: Existing annotated data/manual annotation/automated annotation

1.6 Domain expert for annotation (**Options**: Yes/No/Unspecified)

1.7 Annotator number (if manual annotation performed)

1.8 Inter-annotator agreement (if manual annotation performed)

2. Outcome

2.1 (Built) Clinical Registry

2.2 Registry patient cohort

2.3 Number of the Data elements extracted from free text

2.4 List all the extracted data elements

3. Model development

3.1 Task definition (**Options**: NER/Text classification/NLI/Rule-based NLP/Other)

3.2 Modelling method (algorithm)

4. Model performance

4.1 Evaluation metrics (**Table**: metric names (Recall, precision, F1 and other) /scores)

5. Model evaluation

5.1 Method used for testing model performance

5.1.1 Internal validation (**Options**: Random split/non-random split/cross-validation/Other/No information/Unclear)

5.1.2 External validation (**Options**: Temporal/Geographical/Different setting/Different investigator/Completely independent/Other/No information/Unclear)

6. Results

6.1 Availability of dataset (**Options**: Yes/No/Unspecified)

6.2 Availability of source code (**Options**: Yes/No/Unspecified)

6.3 Availability of trained model (**Options**: Yes/No/Unspecified)

6.4 Implementation of the model (**Options**: Yes/No/Unspecified)

**Appendix 5: Risk of Bias Assessment tool**

To evaluate methodological quality and potential bias of studies using NLP for clinical registry population, we developed a customized risk of bias assessment form derived from the Prediction Model Risk of Bias Assessment Tool (PROBAST) framework. The original PROBAST domains were adapted to address challenges specific to clinical NLP pipelines. The customized form comprises four domains: Data, Modelling, Outcome, and Reproducibility. Each domain includes tailored criteria reflecting key aspects of NLP workflows. For example, the Data domain includes items on sampling strategy, ground truth labelling, and population specificity to reflect the importance of representative and well-annotated clinical text. The Modelling domain assesses whether the NLP task is clearly defined and whether modelling methods are described with sufficient detail, accounting for variation in approaches such as rule-based systems or deep learning. The Outcome domain includes standard performance metrics and distinguishes between internal and external validation, which are particularly relevant for assessing generalizability. The Reproducibility domain addresses the availability of data, source code, and trained models, key factors in ensuring transparency and replicability in modern NLP research.

This customized form is specifically designed to capture methodological nuances of NLP studies that are not addressed by conventional bias assessment tools. Clinical registry population poses distinct informatics challenges compared to other NLP applications such as phenotyping or case detection, including the need to extract structured, longitudinal data across diverse patient records. These tasks require high precision and scalability, making aspects such as task definition, model validation, and data representativeness especially critical. Furthermore, the field of clinical NLP is evolving rapidly, with increasing expectations for reproducibility; the inclusion of reproducibility criteria reflects these standards. By aligning traditional risk-of-bias considerations with the technical and practical realities of NLP-based registry population, the form ensures that studies are assessed in a way that is both rigorous and context-appropriate.

**Risk of Bias Assessment Form**

*(All questions have the options: Yes, No, and Unspecified)*

1. Data **(All unspecified is unsure, all Yes is low risk, otherwise high risk)**

1.1 Sampling: report details of the sampling strategy

1.2 Labelled data: report the ground truth dataset

1.3 Small/specific population

2. Modelling **(All unspecified is unsure, all Yes is low risk, otherwise high risk)**

2.1 Definition of NLP task: clearly define the NLP task

2.2 Modelling method: report details of modelling method

3. Outcome **(All unspecified is unsure, all first 3 questions Yes is low risk, otherwise high risk)**

3.1 Data elements: report details of the extracted data elements from the text

3.2 Performance metrics: report model performance metrics (e.g., F1, recall)

3.3 Internal validation: validate the model performance on the data it was trained on

3.4 External validation: validate the model performance on new and unseen data from different settings

4. Reproducibility **(All unspecified is unsure, all No is high risk, otherwise low risk)**

4.1 Availability of data

4.2 Availability of source code

4.3 Availability of trained model

**Appendix 6: Risk of bias and applicability summary for each study**

| **Study** | **Domain Risk of Bias** | | | | **Overall** | | **Justification** |
| --- | --- | --- | --- | --- | --- | --- | --- |
|  | **Data** | **Modelling** | **Outcome** | **Reproducibility** | **Risk of Bias** | **Applicability** |  |
| Al-Haddad et al. 2010^1^ | High | Low | Low | High | High | Low | 1. No reporting sampling strategy 2. No large population 3. No external validation 4. No availability of data 5. No availability of source code 6. No availability of trained model |
| Davis et al. 2013^2^ | Low | Low | Low | Low | Low | Low | 1. No external validation 2. No availability of data |
| Rastegar-Mojarad et al. 2017^3^ | High | Low | Low | High | High | Low | 1. No reporting sampling strategy 2. No reporting ground truth 3. No external validation 4. No availability of data 5. No availability of source code 6. No availability of trained model |
| Tian et al. 2019^4^ | Low | Low | Low | Low | Low | Low | 1. No external validation 2. No availability of data 3. No availability of trained model |
| Alawad et al. 2021^5^ | Low | Low | Low | High | High | Low | 1. No external validation 2. No availability of data 3. No availability of source code 4. No availability of trained model |
| Percha et al. 2022^6^ | Low | Low | Low | Low | Low | Low | 1. No external validation 2. No availability of data 3. No availability of trained model |
| Cheung et al. 2023^7^ | Low | Low | Low | Low | Low | Low | 1. No external validation 2. No availability of data 3. No availability of trained model |
| Macri et al. 2023^8^ | High | Low | Low | Low | High | Low | 1. No reporting sampling strategy 2. No large population 3. No external validation 4. No availability of data 5. No availability of trained model |
| Barr et al. 2023^9^ | High | Low | High | Low | High | Low | 1. No reporting ground truth 2. No reporting performance metrics 3. No external validation 4. No availability of source code 5. No availability of trained model |
| Raza et al. 2023^10^ | Low | Low | Low | Low | Low | Low | 1. No availability of source code 2. No availability of trained model |
| Bosch et al. 2023^11^ | High | High | Low | High | High | Low | 1. No reporting sampling strategy 2. No reporting ground truth 3. No reporting details of the modelling method 4. No external validation 5. No availability of data 6. No availability of source code 7. No availability of trained model |
| Tavabi et al. 2024^12^ | Low | Low | Low | High | High | Low | 1. No availability of data 2. No availability of source code 3. No availability of trained model |
| Dai et al. 2024^13^ | High | Low | Low | High | High | Low | 1. No large population 2. No availability of data 3. No availability of source code 4. No availability of trained model |
| Lee et al. 2024^14^ | Low | Low | Low | Low | Low | Low | 1. No availability of trained model |
| Mou et al. 2024^15^ | High | Low | Low | Low | High | Low | 1. No large population 2. No external validation 3. No availability of data |

**Appendix 7: Data elements extracted from clinical text for each study**

| **Study** | **Number of data elements** | **Data elements / Data type** |
| --- | --- | --- |
| Al-Haddad et al. 2010^1^ | 1 | Intraductal papillary mucinous neoplasm (IPMN) / Categorical (Binary) |
| Davis et al. 2013^2^ | 8 | Clinical subtype / Categorical (Nominal)  Presence of oligoclonal bands / Categorical (Binary)  Year of diagnosis / Numerical (Discrete)  Expanded Disability Status Scale (EDSS) score / Categorical (Ordinal)  Timed 25-foot walk / Numerical (Continuous)  Year of first neurological symptom / Numerical (Discrete)  Origin of first neurological symptom / Categorical (Nominal)  Multiple sclerosis medications / Categorical (Nominal) |
| Rastegar-Mojarad et al. 2017^3^ | 1 | Procedure / Categorical (Nominal) |
| Tian et al. 2019^4^ | 56 | Heart rate / Numerical (Continuous)  Blood pressure (systolic) / Numerical (Continuous)  Blood pressure (diastolic) / Numerical (Continuous)  Height / Numerical (Continuous)  Weight / Numerical (Continuous)  Typical angina / Categorical (Binary)  Non-chest pain symptoms / Categorical (Binary)  Echocardiographic abnormalities / Categorical (Binary)  Atypical discomfort / Categorical (Binary)  Abnormal ECG / Categorical (Binary)  Is hypertension? / Categorical (Binary)  Highest blood pressure / Numerical (Continuous)  Effect of treatment for hypertension / Categorical (Binary)  Is dyslipidemia? / Categorical (Binary)  Effect of treatment for dyslipidemia / Categorical (Binary)  Is diabetes? / Categorical (Binary)  Type of diabetes / Categorical (Nominal)  Effect of treatment for diabetes / Categorical (Binary)  Is stroke? / Categorical (Binary)  Date of stroke / Categorical (Ordinal)  Place of stroke occurrence / Categorical (Nominal)  Type of stroke / Categorical (Nominal)  Is any peripheral vascular disease? / Categorical (Binary)  Date of peripheral vascular disease / Categorical (Ordinal)  Place of peripheral vascular disease / Categorical (Nominal)  Is myocardial infarction? / Categorical (Binary)  Date of myocardial infarction / Categorical (Ordinal)  History of other diseases / Categorical (Binary)  Status of smoking / Categorical (Nominal)  Number of cigarettes per day / Numerical (Continuous)  Years of smoking / Numerical (Continuous)  Duration of quit smoking (years) / Numerical (Continuous)  Duration of quit smoking (months) / Numerical (Continuous)  Status of drinking / Categorical (Nominal)  Consumption of Liquor at a time (ml) / Numerical (Continuous)  Degree of Liquor / Numerical (Continuous)  Consumption of wine each time (ml) / Numerical (Continuous)  Consumption of rice wine each time (ml) / Numerical (Continuous)  Consumption of beer each time (ml) / Numerical (Continuous)  Duration of quit alcohol / Numerical (Continuous)  Frequency of exercise per week / Numerical (Continuous)  Duration of each workout / Numerical (Continuous)  Current medication of hypertension / Categorical (Nominal)  Current medication of dyslipidemia / Categorical (Nominal)  Current medication of diabetes / Categorical (Nominal)  Current medication / Categorical (Nominal)  Family history of coronary heart disease / Categorical (Binary)  Father has / Categorical (Binary)  Age of father onset / Numerical (Continuous)  Mother has / Categorical (Binary)  Age of mother onset / Numerical (Continuous)  Brothers or Sisters have / Categorical (Binary)  Minimum age of sister or brother onset / Numerical (Continuous)  Children have / Categorical (Binary)  Minimum age of children onset / Numerical (Continuous)  Family history of hyperlipidemia / Categorical (Binary) |
| Alawad et al. 2021^5^ | 6 | Tumor anatomic location site / Categorical (Nominal)  Tumor subsite / Categorical (Nominal)  Tumor laterality / Categorical (Nominal)  Tumor behavior / Categorical (Nominal)  Tumor histology / Categorical (Nominal)  Tumor grade / Categorical (Ordinal) |
| Percha et al. 2022^6^ | 43 | Age / Numerical (Continuous)  Site / Categorical (Nominal)  Reconst / Categorical (Nominal)  Neo_adj / Categorical (Nominal)  Sln_bx_left / Categorical (Binary)  Palpable_right / Categorical (Binary)  Sln_bx_right / Categorical (Binary)  Path_dx / Categorical (Nominal)  Palpable_left / Categorical (Binary)  Genetics / Categorical (Nominal)  Fhx_breast / Categorical (Binary)  Gravida / Numerical (Continuous)  Hormone_use / Categorical (Nominal)  Smoking / Categorical (Nominal)  Margins_right / Categorical (Nominal)  Margins_left / Categorical (Nominal)  Grade_left / Categorical (Ordinal)  Grade_right / Categorical (Ordinal)  Image_biop / Categorical (Nominal)  Para / Numerical (Continuous)  Lvi_right / Categorical (Binary)  Lvi_left / Categorical (Binary)  Status / Categorical (Nominal)  Prin_finding_left / Categorical (Nominal)  Prin_finding_right / Categorical (Nominal)  Surgery_type_left / Categorical (Nominal)  Ax_dissect_right / Categorical (Binary)  Fertility_rx / Categorical (Nominal)  Sln_bx_left_n / Categorical (Binary)  Fhx_ovar / Categorical (Binary)  Surgery_type_right / Categorical (Nominal)  Sln_bx_right_n / Categorical (Binary)  Ax_dissect_left / Categorical (Binary)  Prior_surg / Categorical (Binary)  Ax_dissect_right_n / Categorical (Binary)  Personal_hx / Categorical (Nominal)  Ax_dissect_left_n / Categorical (Binary)  Er_right / Categorical (Binary)  Er_left / Categorical (Binary)  Her2_left / Categorical (Binary)  Her2_right / Categorical (Binary)  Pr_left / Categorical (Binary)  Pr_right / Categorical (Binary) |
| Cheung et al. 2023^7^ | 14 | Corpectomy / Categorical (Nominal)  ACDF (Anterior Cervical Discectomy and Fusion) / Categorical (Nominal)  ALIF (Anterior Lumbar Interbody Fusion) / Categorical (Nominal)  AxiaLIF (Axial Lumbar Interbody Fusion) / Categorical (Nominal)  Fusion / Categorical (Nominal)  Laminectomy / Categorical (Nominal)  LLIF (Lateral Lumbar Interbody Fusion) / Categorical (Nominal)  Osteotomy / Categorical (Nominal)  PLIF (Posterior Lumbar Interbody Fusion) / Categorical (Nominal)  TLIF (Transforaminal Lumbar Interbody Fusion) / Categorical (Nominal)  VCR (Vertebral Column Resection) / Categorical (Nominal)  Vertebrectomy / Categorical (Nominal)  Vertebroplasty / Categorical (Nominal)  XLIF (Extreme Lateral Lumbar Interbody Fusion) / Categorical (Nominal) |
| Macri et al. 2023^8^ | 1 | Diagnosis / Categorical (Nominal) |
| Barr et al. 2023^9^ | 38 | Personal Health Identification – Categorical (Nominal)  Last name / Categorical (Nominal)  First name / Categorical (Nominal)  Date of birth / Categorical (Ordinal)  Age / Numerical (Continuous)  Date biopsy collected / result received / Categorical (Ordinal)  Diagnosis / Categorical (Nominal)  Cores / Numerical (Continuous)  Total glomeruli / Numerical (Continuous)  Sclerosed glomeruli / Numerical (Continuous)  Glomeruli with segmental sclerosis / Numerical (Continuous)  Glomeruli with fibrinoid necrosis / Numerical (Continuous)  Glomeruli with cellular crescents / Numerical (Continuous)  Glomeruli with fibrocellular crescents / Numerical (Continuous)  Glomeruli with fibrous crescents / Numerical (Continuous)  IFTA (interstitial fibrosis and tubular atrophy) / Numerical (Continuous)  Intimal fibrosis / Numerical (Continuous)  Vasculitis / Categorical (Binary)  Immunofluorescence / Categorical (Nominal)  IgG / Numerical (Continuous)  IgA / Numerical (Continuous)  IgM / Numerical (Continuous)  AHIg / Numerical (Continuous)  C3 / Numerical (Continuous)  C1q / Numerical (Continuous)  Fibrinogen / Numerical (Continuous)  Kappa / Numerical (Continuous)  Lambda / Numerical (Continuous)  Foot Process Effacement (FPE) / Categorical (Binary)  Subepithelial deposits / Categorical (Binary)  Subendothelial deposits / Categorical (Binary)  Intramembranous deposits / Categorical (Binary)  Mesangial deposits / Categorical (Binary)  PLA2R / Categorical (Binary)  Oxford M/E/S/T/C / Categorical (Nominal)  Lupus class / Categorical (Nominal)  Lupus AI (activity index) / Numerical (Continuous)  Lupus CI (chronicity index) / Numerical (Continuous) |
| Raza et al. 2023^10^ | 44 | Gender / Categorical (Nominal)  Height / Numerical (Continuous)  Age / Numerical (Continuous)  Date / Categorical (Ordinal)  Weight / Numerical (Continuous)  Smoking / Categorical (Nominal)  Race_ethnicity / Categorical (Nominal)  Relative_date / Categorical (Ordinal)  Drug_name / Categorical (Nominal)  Duration / Numerical (Continuous)  Admission_discharge / Categorical (Nominal)  Alcohol / Categorical (Nominal)  Substance / Categorical (Nominal)  Employment / Categorical (Nominal)  Time / Numerical (Continuous)  Oxygen_therapy / Categorical (Binary)  Heart_disease / Categorical (Binary)  Clinical_department / Categorical (Nominal)  Blood_pressure / Numerical (Continuous)  Disease_syndrome / Categorical (Nominal)  Dosage / Numerical (Continuous)  Treatment / Categorical (Nominal)  Test / Categorical (Nominal)  Psychological_condition / Categorical (Nominal)  Symptom / Categorical (Nominal)  Respiration / Categorical (Nominal)  Labour_delivery / Categorical (Nominal)  Internal_organ / Categorical (Nominal)  External_body_part / Categorical (Nominal)  Procedure / Categorical (Nominal)  Diabetes / Categorical (Binary)  Vaccine / Categorical (Nominal)  Hyperlipidemia / Categorical (Binary)  Hypertension / Categorical (Binary)  Death_entity / Categorical (Nominal)  Symptom / Categorical (Nominal)  Respiration / Categorical (Nominal)  Labour_delivery / Categorical (Nominal)  Temperature / Numerical (Continuous)  Kidney_disease / Categorical (Binary)  Obesity / Categorical (Binary)  Bmi / Numerical (Continuous)  Pulse / Numerical (Continuous)  Injury_or_poisoning / Categorical (Nominal) |
| Bosch et al. 2023^11^ | 11 | Date of initial diagnosis / Categorical (Ordinal)  Type of tumor / Categorical (Nominal)  Gleason score / Numerical (Discrete)  Weight / Numerical (Continuous)  ECOG PS (Eastern Cooperative Oncology Group Performance Status) / Categorical (Ordinal)  PSA (Prostate-Specific Antigen) / Numerical (Continuous)  Hb (Hemoglobin) / Numerical (Continuous)  MDT-date (Multidisciplinary Team date) / Categorical (Ordinal)  Drug treatment / Categorical (Nominal)  Start date of drug / Categorical (Ordinal)  Dose of drug / Numerical (Continuous) |
| Tavabi et al. 2024^12^ | 18 | Laterality / Categorical (Nominal)  Revision / Categorical (Binary)  Graft Type / Categorical (Nominal)  Interference / Categorical (Nominal)  Endobutton / Categorical (Binary)  Tightrope / Categorical (Binary)  Medial Meniscus / Categorical (Binary)  Lateral Meniscus / Categorical (Binary)  Laterality / Categorical (Nominal)  Injury Mech (Injury Mechanism) / Categorical (Nominal)  Tear Type / Categorical (Nominal)  Skeletal Age / Numerical (Continuous)  Ipsilateral ACL reinjury / Categorical (Binary)  MCL (Medial collateral ligament) Injury / Categorical (Binary)  Lateral meniscus injury / Categorical (Binary)  Medical meniscus injury / Categorical (Binary)  PCL (Posterior Cruciate Ligament) Injury / Categorical (Binary)  PLC ( Posterolateral corner) Injury / Categorical (Binary) |
| Dai et al. 2024^13^ | 30 | AJCC Edition / Categorical (Ordinal)  Date of First Surgical Procedure / Categorical (Ordinal)  Diagnostic Confirmation / Categorical (Nominal)  Date of Initial Diagnosis / Categorical (Ordinal)  Other Staging System / Categorical (Nominal)  Clinical Other Staging Group / Categorical (Nominal)  SSF2 / Categorical (Ordinal)  Laterality / Categorical (Nominal)  Date of First Microscopic Confirmation / Categorical (Ordinal)  Pathologic Stage Descriptor / Categorical (Nominal)  SSF7 / Categorical (Ordinal)  Surgical Margins / Categorical (Ordinal)  Behavior Code / Categorical (Nominal)  Grade Pathological / Categorical (Ordinal)  Pathologic M / Categorical (Ordinal)  Pathologic N / Categorical (Ordinal)  Scope of Regional Lymph Node Surgery / Categorical (Ordinal)  Pathologic T / Categorical (Ordinal)  Clinical Stage Descriptor / Categorical (Nominal)  Grade Clinical / Categorical (Ordinal)  Nodes Positive / Numerical (Discrete)  Primary Site / Categorical (Nominal)  Nodes Examined / Numerical (Discrete)  Perineural Invasion / Categorical (Binary)  Histology / Categorical (Nominal)  Date of Surgical Diagnostic and Staging Procedure / Categorical (Ordinal)  Lymph vessels or Vascular Invasion / Categorical (Binary)  SSF6 / Categorical (Ordinal)  Surgical Margins Distance / Numerical (Continuous)  SSF5 / Categorical (Ordinal) |
| Lee et al. 2024^14^ | 94 | SP_TV_Native / Categorical (Binary)  SP_MV_Native / Categorical (Binary)  SP_Cardiac_Valve_Any / Categorical (Binary)  SP_MV_Surgery / Categorical (Binary)  SP_CABG / Categorical (Binary)  SP_AV_Surgery / Categorical (Binary)  SP_MV_Replacement / Categorical (Binary)  SP_AV_Replacement / Categorical (Binary)  SP_TV_Surgery / Categorical (Binary)  SP_PV_Replacement / Categorical (Binary)  SP_MV_Repair / Categorical (Binary)  SP_AV_Native / Categorical (Binary)  SP_TAVR / Categorical (Binary)  SP_MV_Annuloplasty / Categorical (Binary)  SP_MV_Implant_Type_Annuloplasty_Device / Categorical (Binary)  SP_TV_Repair / Categorical (Binary)  SP_TV_Annuloplasty / Categorical (Binary)  SP_Aorta_Surgery / Categorical (Binary)  SP_Aorta_Major_Proc / Categorical (Binary)  SP_AV_Implant_Type_Bioprosthesis / Categorical (Binary)  SP_Aortic_Root_Asc_Or_Arch / Categorical (Binary)  SP_Afib_Procedure_Any / Categorical (Binary)  SP_Other_Cardiac / Categorical (Binary)  SP_MV_Implant_Type_Bioprosthesis / Categorical (Binary)  SP_Any_Mech_Assist_Insertion / Categorical (Binary)  SP_MV_Leaflet_Resection / Categorical (Binary)  SP_AV_Explant / Categorical (Binary)  SP_AV_Bentall / Categorical (Binary)  SP_Mech_Assist_Implant_Or_Removal / Categorical (Binary)  SP_MV_Transcatheter_Replacement / Categorical (Binary)  SP_PV_Surgery / Categorical (Binary)  SP_MV_Explant / Categorical (Binary)  SP_Removal_of_Atrial_Myxoma_Cardiac_Tumors / Categorical (Binary)  SP_Heart_Transplant / Categorical (Binary)  SP_Septal_Myectomy / Categorical (Binary)  SP_TV_Replacement / Categorical (Binary)  SP_VAD3_Implant / Categorical (Binary)  SP_AV_Commissural_Annuloplasty / Categorical (Binary)  SP_Pulmonary_Thromboembolectomy / Categorical (Binary)  SP_Valve_Sparing_Root_Reimplantation_David / Categorical (Binary)  SP_TV_Explant / Categorical (Binary)  SP_Descending_Aorta / Categorical (Binary)  SP_AV_Leaflet_Commissural_Resuspension_suture / Categorical (Binary)  SP_AV_Repair / Categorical (Binary)  SP_Pericardiectomy / Categorical (Binary)  SP_VAD2_Implant / Categorical (Binary)  SP_AV_Implant_Type_Homograft / Categorical (Binary)  SP_MV_Neochords_PTFE / Categorical (Binary)  SP_Carotid_Endarterectomy / Categorical (Binary)  SP_IABP_Insertion / Categorical (Binary)  SP_VAD_Previously_Implanted / Categorical (Binary)  SP_AV_Leaflet_Plication / Categorical (Binary)  SP_AV_Implant_Type_Autograft_Ross / Categorical (Binary)  SP_MV_Implant_Type_Mechanical / Categorical (Binary)  SP_Non_Cardiac / Categorical (Binary)  SP_AV_Division_of_Fused_Leaflet_Raphe / Categorical (Binary)  SP_AV_Leaflet_Pericardial_Patch / Categorical (Binary)  SP_AV_Implant_Type_Mechanical / Categorical (Binary)  SP_MV_Cleft_Repair_Scallop_Closure / Categorical (Binary)  SP_MV_Repair_Attempt / Categorical (Binary)  SP_Other_Congenital / Categorical (Binary)  SP_PV_Repair / Categorical (Binary)  SP_MV_Implant_Type_Other / Categorical (Binary)  SP_Subaortic_Stenosis_Resection / Categorical (Binary)  SP_ECMO_Insertion / Categorical (Binary)  SP_AV_Leaflet_Debridement / Categorical (Binary)  SP_MV_Edge_to_Edge_Repair / Categorical (Binary)  SP_Other_Vascular_Procedure / Categorical (Binary)  SP_Cath_Based_Assist_Device_Used / Categorical (Binary)  SP_AV_Annular_Enlargement / Categorical (Binary)  SP_MV_Leaflet_Plication / Categorical (Binary)  SP_MV_Sliding_Plasty / Categorical (Binary)  SP_Ventricular_Septal_Defect_Repair / Categorical (Binary)  SP_MV_Commissuroplasty / Categorical (Binary)  SP_MV_Commissurotomy / Categorical (Binary)  SP_ASD_PFO_Repair / Categorical (Binary)  SP_Other_Thoracic_Procedure / Categorical (Binary)  SP_AV_Leaflet_Free_Edge_Reinforcement / Categorical (Binary)  SP_AV_Ring_Annuloplasty / Categorical (Binary)  SP_MV_Leaflet_Extension_Replacement_Patch / Categorical (Binary)  SP_Any_Major_Ventricular_Procedure / Categorical (Binary)  SP_MV_Chordal_Leaflet_Transfer / Categorical (Binary)  SP_MV_Annular_Decalcification_Debridement / Categorical (Binary)  SP_Pericardial_Window / Categorical (Binary)  SP_Valve_Sparing_Root_remodeling_Yacoub / Categorical (Binary)  SP_Valve_Sparing_Resuspension_Asc_Ao / Categorical (Binary)  SP_Valve_Sparing_Root_Reconstruction_Florida / Categorical (Binary)  SP_AV_Leaflet_Resection_Suture / Categorical (Binary)  SP_AV_Repair_of_Periprosthetic_Leak / Categorical (Binary)  SP_Transmyocardial_Laser_Revascularization / Categorical (Binary)  SP_Trauma_Repair / Categorical (Binary)  SP_Valve_Sparing_Resuspension_No_Asc_Ao / Categorical (Binary)  SP_MV_Native_Pannus_Thrombus_Removal / Categorical (Binary)  SP_MV_Prosthetic_Valve_Repair / Categorical (Binary) |
| Mou et al. 2024^15^ | 19 | Examination Date / Categorical (Ordinal)  Submission Number / Numerical (Continuous)  Examined Preparation / Categorical (Nominal)  Type of Biopsy / Categorical (Nominal)  Biopsy Sampling Site / Categorical (Nominal)  Localization / Categorical (Nominal)  Tumor Proof / Categorical (Binary)  ICD-O3 History / Categorical (Nominal)  Grading / Categorical (Ordinal)  Estrogen Positive Cells / Numerical (Continuous)  Estrogen Intensity / Categorical (Ordinal)  Estrogen Score / Categorical (Ordinal)  Progesterone Positive Cells / Numerical (Continuous)  Progesterone Intensity / Categorical (Ordinal)  Progesterone Score / Categorical (Ordinal)  HER2 / Categorical (Ordinal)  Ki-67 / Numerical (Continuous)  ICD-10 Diagnosis / Categorical (Nominal)  Side / Categorical (Nominal) |

Notes: ACDF: anterior cervical discectomy & fusion; ALIF: anterior lumbar interbody fusion; AxiaLIF: axial lumbar interbody fusion; LLIF: lateral lumbar interbody fusion; PLIF: posterior lumbar interbody fusion; TLIF: transforaminal lumbar interbody fusion; VCR: vertebral column resection; XLIF: extreme (far) lateral interbody fusion; ECOG PS: Eastern Cooperative Oncology Group Performance Status; PSA: Prostate-Specific Antigen; Hb: Hemoglobin; MDT-date: Multi-Disciplinary Team date

**Appendix 8: Availability and implementation of the studies**

| **Study** | **Availability of data** | **Availability of source code** | **Availability of trained model/rules** | **Implementation** |
| --- | --- | --- | --- | --- |
| Al-Haddad et al. 2010^1^ |  |  |  |  |
| Davis et al. 2013^2^ |  | Yes | Yes |  |
| Rastegar-Mojarad et al. 2017^3^ |  |  |  |  |
| Tian et al. 2019^4^ |  | Yes |  | Yes |
| Alawad et al. 2021^5^ |  |  |  |  |
| Percha et al. 2022^6^ |  | Yes |  |  |
| Cheung et al. 2023^7^ |  | Yes |  |  |
| Macri et al. 2023^8^ |  | Yes |  |  |
| Barr et al. 2023^9^ |  |  |  | Yes |
| Raza et al. 2023^10^ | Yes |  |  |  |
| Bosch et al. 2023^11^ |  |  |  | Yes |
| Tavabi et al. 2024^12^ |  |  |  | Yes |
| Dai et al. 2024^13^ |  |  |  | Yes |
| Lee et al. 2024^14^ | Yes | Yes |  |  |
| Mou et al. 2024^15^ |  | Yes | Yes |  |

*Notes: If left Blank, it means no.*

**References**

1. Al-Haddad MA, Friedlin J, Kesterson J, et al. Natural language processing for the development of a clinical registry: a validation study in intraductal papillary mucinous neoplasms. *HPB (Oxford)* 2010;12(10):688-95. doi: 10.1111/j.1477-2574.2010.00235.x

2. Davis MF, Sriram S, Bush WS, et al. Automated extraction of clinical traits of multiple sclerosis in electronic medical records. *JOURNAL OF THE AMERICAN MEDICAL INFORMATICS ASSOCIATION* 2013;20(E2):E334-E40. doi: 10.1136/amiajnl-2013-001999

3. Rastegar-Mojarad M, Sohn S, Wang L, et al. Need of informatics in designing interoperable clinical registries. *Int J Med Inform* 2017;108:78-84. doi: 10.1016/j.ijmedinf.2017.10.004

4. Tian Q, Liu M, Min L, et al. An automated data verification approach for improving data quality in a clinical registry. *Comput Methods Programs Biomed* 2019;181:104840. doi: 10.1016/j.cmpb.2019.01.012

5. Alawad M, Yoon HJ, Gao S, et al. Privacy-Preserving Deep Learning NLP Models for Cancer Registries. *IEEE Transactions on Emerging Topics in Computing* 2021;9(3):1219-30. doi: 10.1109/TETC.2020.2983404

6. Percha B, Pisapati K, Gao C, et al. Natural language inference for curation of structured clinical registries from unstructured text. *Journal of the American Medical Informatics Association* 2022;29(1):97-108. doi: https://dx.doi.org/10.1093/jamia/ocab243

7. Cheung ATM, Kurland DB, Neifert S, et al. Developing an Automated Registry (Autoregistry) of Spine Surgery Using Natural Language Processing and Health System Scale Databases. *Neurosurgery* 2023;93(6):1228-34. doi: 10.1227/neu.0000000000002568

8. Macri CZ, Teoh SC, Bacchi S, et al. A case study in applying artificial intelligence-based named entity recognition to develop an automated ophthalmic disease registry. *Graefes Arch Clin Exp Ophthalmol* 2023;261(11):3335-44. doi: 10.1007/s00417-023-06190-2

9. Barr B, Harasemiw O, Gibson IW, et al. The Development of a Comprehensive Clinicopathologic Registry for Glomerular Diseases Using Natural Language Processing. *Can J Kidney Health Dis* 2023;10:20543581231178963. doi: 10.1177/20543581231178963

10. Raza S, Schwartz B. Constructing a disease database and using natural language processing to capture and standardize free text clinical information. *Sci Rep* 2023;13(1):8591. doi: 10.1038/s41598-023-35482-0

11. Bosch D, Kuppen MCP, Tascilar M, et al. Reliability and Efficiency of the CAPRI-3 Metastatic Prostate Cancer Registry Driven by Artificial Intelligence. *Cancers (Basel)* 2023;15(15) doi: 10.3390/cancers15153808

12. Tavabi N, Pruneski J, Golchin S, et al. Building large-scale registries from unstructured clinical notes using a low-resource natural language processing pipeline. *Artif Intell Med* 2024;151:102847. doi: 10.1016/j.artmed.2024.102847

13. Dai HJ, Chen CC, Mir TH, et al. Integrating predictive coding and a user-centric interface for enhanced auditing and quality in cancer registry data. *COMPUTATIONAL AND STRUCTURAL BIOTECHNOLOGY JOURNAL* 2024;24:322-33. doi: 10.1016/j.csbj.2024.04.007

14. Lee J, Sharma I, Arcaro N, et al. Automating surgical procedure extraction for society of surgeons adult cardiac surgery registry using pretrained language models. *JAMIA Open* 2024;7(3):ooae054. doi: 10.1093/jamiaopen/ooae054

15. Mou Y, Lehmkuhl J, Sauerbrunn N, et al. Improving the Quality of Unstructured Cancer Data Using Large Language Models: A German Oncological Case Study. *Studies in health technology and informatics* 2024;316((Mou, Lehmkuhl, Sowe, Decker) Chair of Computer Science 5, RWTH Aachen University, Germany(Lehmkuhl, Sowe, Decker) Fraunhofer FIT, Germany(Sauerbrunn, Kochel, Panse) Center for Integrated Oncology, University Hospital Aachen, Germany(Panse, Brummendorf) D):685EP-89. doi: https://dx.doi.org/10.3233/SHTI240507
